# Preparation of RGO and Anionic Polyacrylamide Composites for Removal of Pb(II) in Aqueous Solution

**DOI:** 10.3390/polym12061426

**Published:** 2020-06-26

**Authors:** Lili Wu, Mengke Li, Mu Li, Qiyue Sun, Chaocan Zhang

**Affiliations:** School of Materials Science and Engineering, Wuhan University of Technology, Wuhan 430070, China; 1049721800235@whut.edu.cn (M.L.); 265860@whut.edu.cn (M.L.); sunqiyue93@163.com (Q.S.)

**Keywords:** graphene oxide, ethylenediamine, anionic polyacrylamide, adsorbent

## Abstract

Graphene oxide (GO) have been reported as adsorbent materials, because its surface contains a large number of oxygen-containing groups, which provide masses of active sites. Nevertheless, it is difficult to separate GO from aqueous solution by conventional means after the end of the adsorption process. Therefore, ethylene diamine-reduced graphene oxide/anionic polyacrylamide (E-RGO/APAM), with a large quantity of adsorption sites and strong flocculation was prepared in this study. The composite E-RGO/APAM was characterized by Fourier transform infrared (FTIR), laser Raman spectrometer (IR), scanning electron microscope (SEM). The obtained results indicated that amino groups were successfully introduced into GO. Particle size test showed that the particle size of E-RGO/APAM is up to three micrometers, which can be separated from the water by conventional means, such as filtration and centrifugation, to avoid secondary pollution. The efficiency of E-RGO/APAM for removing Pb(II) was tested. The results showed that the process of adsorption of Pb(II) by E-RGO/APAM can be fitted by pseudo second order kinetic equation, indicating that the adsorption rate of the adsorbent depends on the chemisorption process, and the theoretical maximum adsorption amount of E-RGO/APAM is 400.8 mg/g. Based on these results, it can be stated that E-RGO/APAM is effective in the removal of Pb(II) from aqueous solutions, and provides a new method for the removal of heavy metal ions from industrial wastewater.

## 1. Introduction

Heavy metal pollution must be attached great importance to, as it contributes tremendously to the damage to the ecology and harm to the human body. Heavy metal ions contained in industrial wastewater diffuse rapidly with water and do not degrade themselves, making them a worldwide problem. Lead is one of the earliest heavy metals used by humans and one of the most heavily used heavy metals, usually from industrial and agricultural wastewater and acidic leachate from landfills [1,2,3]. Increasingly wide range of lead pollution has been occurring because of the continuous development of modern industry, especially the wide application of lead-containing batteries. If the waste water containing heavy metal lead ions is discharged without treatment, it will lead to serious environmental pollution and difficult to repair. Hence, the removal of heavy metal lead ions from industrial wastewater is very important for controlling environmental pollution [4,5,6,7]. Many techniques have been applied in the removal of Pb(II), such as chemical precipitation, exchanging ions, membrane filtration, reverse osmosis, adsorption, etc. Among these techniques, the adsorption method is widely used due to its economic and efficiency [8]. It is well known that scientists have done much research on activated carbon (AC) adsorbent. Fiol et al. [9] prepared AC from olive stone waste generated in olive oil production as a metal biosorbent for Pb^2+^, Ni^2+^, Cu^2+^ and Cd^2+^ from aqueous solution. The maximum adsorption capacities of the Langmuir model were 6.88, 4.47, 3.63 and 3.19 × 10^−5^ mol/g for Cd^2+^, Pb^2+^, Ni^2+^ and Cu^2+^, respectively. Park et al. [10] prepared an activated carbon-containing alginate bead (AC–AB) adsorbent, which successfully used to simultaneously remove heavy metal ions and toxic organics. A 50-L solution containing a variety of heavy metal ions and mineral ions was run continuously through a filter cartridge packed with 160 g of the AC–AB adsorbent. The adsorbent showed a high capacity to remove heavy metal ions completely from the water, while allowing essential minerals to pass through the filter. Ge et al. [11] synthesized a novel activated carbon–chitosan complex adsorbent (ACCA). The results demonstrated that ACCA has higher adsorption capacity than chitosan.

Graphene has been a source for scientific inspiration because of its supreme material parameters. Graphene-based adsorbents have attracted extensive attention in the field of wastewater treatment. Graphene oxide (GO) is a kind of single- or multilayer nanomaterial formed by the fracture of interlamellar bonds of graphite under the action of strong oxidant, which has many similarities with the structure of graphene [12,13,14,15,16]. What is more, compared with graphene, GO contains a large number of oxygen-containing groups on its surface, which not only provides a large number of active sites on its surface, but also improves its hydrophilicity, thus greatly improving its adsorption performance to heavy metal ions [17,18]. In order to obtain higher adsorption efficiency of graphene-based adsorbent, many studies from literature have reported functional modification of graphene through covalent or non-covalent action [19,20,21]. The grafts of sulfanilic acid, ethylenediamine tetraacetic acid (EDTA) and ethylenediamine (ED) on the surface of GO can be used to prepare corresponding composites to increase surface area and adsorb functional groups. It is worth pointing out that the amino group in ED can combine with heavy metal ions such as copper and lead ions in water to form complex with high stability constant. Madadrang et al. [22] prepared EDTA–GO by covalent reaction. The analysis showed a significant increase in the number of functional groups, and the adsorption capacity of Pb(II) increased from 328 ± 39 mg/g to 479 ± 46 mg/g. Fu et al. [23] synthesized dithiocarbamate(DTC) modified magnetic reduce graphene oxide (RGO-PDTC/Fe3O4). The results indicated that it is an excellent adsorbent for heavy metal ions removal, which exhibits large adsorption capacities, fast kinetics and solid–liquid separation. The maximum adsorption capacities of the Langmuir model were 113.64, 116.28, 147.06 and 181.82 mg/g for Cu(II), Cd(II), Pb(II) and Hg(II) ions, respectively. Fathima Arshad et al. [24] synthesized polyethylenimine modified graphene oxide hydrogel composite. The maximum uptake of heavy metal ions was 602, 374 and 181 mg/g for Pb (II), Hg (II) and Cd (II) ions, respectively. Wu et al. [25] synthesized cetyltrimethylammonium bromide-modified graphene (CTAB) by non-covalent bond, which effectively prevented the agglomeration of graphene and maintained the inherent properties of graphene. Unfortunately, it is easy to cause secondary pollution in the aqueous solution, because of the highly hydrophilic and small size of the graphene oxide.

In order to overcome such shortcoming, GO and Anionic polyacrylamide (APAM) complexes were first prepared in this article. APAM has functional groups with adsorption properties such as an amide group or a carboxyl group, and exhibits an electronegativity, which can lower the potential of the metal cation, and provide favorable ion adsorption capacity [26,27,28]. APAM can bridge between particles in water to concentrate particles and form floc deposits, which allows it to be used as a flocculant [29]. Due to the introduction of active functional groups of APAM, the surface area and porous structure of the complex were improved, and the volume of the complex was increased, which significantly improved its dispersion and stability. Then, ED was used to reduce and modify the GO in the complex. The reduction degree of GO in the complex was controlled by the amount of ED, so that a large number of oxygen-containing functional groups were retained, and amino functional groups were introduced to improve the removal ability of heavy metal ions. Finally, the composite adsorbent ethylenediamine-reduced graphene oxide/anionic polyacrylamide (E-RGO/APAM) was obtained. The aim of this study is to test the performance of E-RGO/APAM composite for the removal of heavy metal Pb(II) ions in aqueous solution. Detailed studies were conducted to test the adsorption efficiency of the functionalized composite E-RGO/APAM compared with the non-functionalized GO-APAM. The effects of modification on specific surface area, surface morphologies, chemical compositions, adsorption mode, and adsorption properties of the composites were systematically investigated. The equilibrium and kinetic modeling of experimental data indicated that the composite E-RGO/APAM has high efficiency in the heavy metal Pb(II) ions removal processes and could be a viable alternative for the industrial wastewater treatment.

## 2. Materials and Methods

### 2.1. Materials

Graphite flakes (325 mesh), concentrated sulfuric acid (98%), concentrated phosphoric acid (85%), hydrogen peroxide (30%), concentrated hydrochloric acid (35.5%–36%), barium chloride (AR), hydrazine hydrate (AR, 85%), ethylenediamine (AR), Potassium permanganate (AR), Lead nitrate (AR), Sodium chloride (AR), Sodium hydroxide (AR) were purchased from Sinopharm chemical reagent factory. Anionic polyacrylamide (industrial grade) was purchased from Wuxi environmental protection technology development co. LTD.

### 2.2. Preparation of GO, GO/APAM, E-RGO, E-RGO/APAM

Graphene oxide(GO) were prepared by the improved synthesis method that Daniela reported in 2010, with graphite is oxidized by treatment with KMnO_4_ in a 9:1 mixture of H_2_SO_4_/H_3_PO_4_ [30]. Afterwards, the aqueous anionic polyacrylamide (APAM) solution was poured into graphene oxide colloidal suspension and mixed for 2 h by a constant temperature oscillator at 40 °C to obtain GO/APAM. The ratios of GO and APAM are 3:1.

A certain amount of ethylenediamine (ED) was added to 100 mL graphene oxide (1 mg/mL) suspension prepared in the previous step and stir magnetically at 80 °C for 3 h. The amount of ED added was 0.1 mL, 0.5 mL and 1 mL, and the corresponding reduced graphene oxides were E-RGO1, E-RGO2 and E-RGO3.

To investigate the characteristic of ethylenediamine-reduced graphene oxide/anionic polyacrylamide (E-RGO/APAM), ethylenediamine with different ratio (0.1 mL, 0.5 mL, 1.0 mL) were poured into GO/APAM suspension and mixed for 3 h by magnetic stirring at 80 °C. After separation by centrifuge, the precipitate was washed with deionized water to neutral. With the different content of ethylenediamine, the corresponding composites were marked as E-RGO/APAM1, E-RGO/APAM2 and E-RGO/APAM3, respectively.

### 2.3. Characterization

SEM (JSM-IT300, Japan) operating at an acceleration voltage 10 kV was used to analyze morphology and the structure of the composites. Before SEM analysis, the samples were ground into powder and dried under vacuum for 24 h. Raman scattering was performed on a laser Raman spectrometer (RENISHAW, Wotton-under-Edge, UK) at 633-nm laser excitation of a He–Ne laser. Attenuated total reflectance Fourier-transform infrared spectroscopy (ATR-FTIR, Nicolet 6700, Thermo Fisher, Waltham, MA, USA) was used to characterize the structure of the composites. The surface areas of the composites were obtained using the Langmuir single-layer adsorption technique. Absorption spectra were analyzed in a UV-Vis spectrophotometer (UV-2550, Shimadzu, Japan). The particle size was determined by nano series dynamic light scattering (DLS Malvern instruments Co, UK). The dynamic light scattering was measured at an angle of 173°. A He–Ne power laser was used, operating at a wavelength of 633 nm and a voltage of 22 mV.

### 2.4. Methylene Blue Adsorption Experiments to Determine the Specific Surface Area

The method for determining the specific surface area of graphene in the laboratory includes nitrogen adsorption method, methylene blue test adsorption method, etc. Among them, the methylene blue test adsorption method is widely used due its advantages of simple operation and accurate results [31]. The methylene blue test adsorption method utilizes the Langmuir monolayer adsorption theory.

The operation of the methylene blue test adsorption method was as follows: 75 mg of methylene blue powder was placed in a 250-mL volumetric flask, and the volume was adjusted by adding deionized water. The solution was diluted to 2.5 mg/L, 5 mg/L, 10 mg/L, 15 mg/L, 20-mg/L and 25 mg/L, and a certain amount of methylene blue solution was pipetted into a quartz cuvette. Using a UV spectrophotometer, wavelength ranges of 200 nm to 800 nm were selected for scanning to determine the wavelength at which the maximum absorbance was obtained. A total of 25 mL of 200-mg/L methylene blue test solution was prepared and used with GO, E-RGO and E-RGO/APAM as adsorbents to adsorb the test solution. This was kept for 24 h in a constant temperature water bath at 25 °C to achieve adsorption equilibrium. The supernatant was filtered through a 0.22-μm pore-size micropore filter and the filtrate was placed in a quartz cuvette. Water was used as a reference solution to measure the absorbance. According to the Beer–Lambert law, when the incident light was monochromatic light of fixed wavelength, the absorbance of the solution was proportional to the concentration of the colored substance in the solution. The concentration of methylene blue could be calculated from the absorbance of the solution after adsorption, and then the adsorption amount (*q,* mg/g) of the adsorbent to methylene blue could be calculated by the following equation:

(1) $$q=\frac{\left(c_{0}-c\right)\times v}{w}$$

where *c_0_* (mg/L) and *c* (mg/L) are the concentration of the original methylene blue and the methylene blue after adsorption in the solution, *v* (L) is the volume of methylene blue and *w* (g) is the weight of the adsorbent. The adsorption amount *q* of the adsorbent is calculated and then the specific surface area (*S*, m^2^/g) of the adsorbent can be calculated by the following equation:

(2) S=q×a

where *a* (m^2^) is the area of the adsorbent coated with 1 mg of methylene blue.

Seen from the ultraviolet-visible absorption spectroscopy, methylene blue has a wavelength of 664 nm at the maximum absorbance and then a series of methylene blue solutions of known concentration are arranged to measure the absorbance at this wavelength. The methylene blue concentration can be obtained by the following equation:

(3) y=0.1375x+0.1431

where *y* is absorbance, *x* is the concentration of methylene blue.

The specific surface area of the adsorbent was obtained by using the equations, and the results showed that the specific surface areas of GO, E-RGO and E-RGO/APAM were 1039, 1292 and 1380 m^2^/g, respectively. The addition of ethylenediamine modified RGO to increase the adsorption sites and adsorption capacity, and APAM was inserted between the RGO sheets to further increase the specific surface area. Moreover, APAM has highly adsorbed amide functional groups, which made E-RGO/APAM had the strongest adsorption capacity for methylene blue, and the test results showed that the specific surface area of E-RGO/APAM was the largest.

### 2.5. Pb(II) Adsorption Experiments

We placed 50 mL of 100-mg/L Pb(II) solution into a 150-mL conical flask, in order to reduce the effect of ions concentration change, added 10 mL 0.1-M NaCl to the solution, adjust the pH with 0.1-mol/L HCl or NaOH, then accurately weighed 0.020 g of dried E-RGO/APAM or GO/APAM into a conical flask. The conical flask was shaken in a constant temperature water bath oscillator for 5 h, the supernatant was filtered through a 0.22-μm microporous membrane after adsorption. The remaining Pb(II) solution concentration was measured by atomic absorption spectrometry, and the adsorption amount (*Qe*, mg/g) of the adsorbent was calculated by the following equation:

*Q_e_* = (*c_0_ −c _e_*) × *v/w* (4)

where *c_0_* (mg/L) and *c_e_* (mg/L) are the concentration of the Pb(II) before and after adsorption in the solution, *v* (L) is the volume of the solution and *w*(g) is the weight of added adsorbent.

The individual effect of single factor was performed under constant conditions of other factors. The influence of ethylenediamine dose on the adsorption performance was investigated at a dose range from 0.1 mL to 1.5 mL. The pH was adjusted to 5.0, and the conical flask was shaken at 30 °C in a constant temperature water bath oscillator for 5 h, and then sampled and tested. The effect of pH was performed at constant ethylenediamine dose of 0.5 mL and the pH range of 2.0 to 6.0. The influences of pH > 6.0 were not studied to avoid the formation of precipitate [11]. The conical flask was shaken at 30 °C in a constant temperature water bath oscillator for 5 h, and then sampled and tested. In order to study the effect of temperature on adsorption capacity, the temperature was changed from 15 °C to 45 °C. The pH was adjusted to 5.0, and the dose of ethylene diamine was 0.5 mL.

## 3. Results and Discussion

### 3.1. Characterization of E-RGO/APAM Composites

#### 3.1.1. Particle Size Analysis

**Figure 1:**
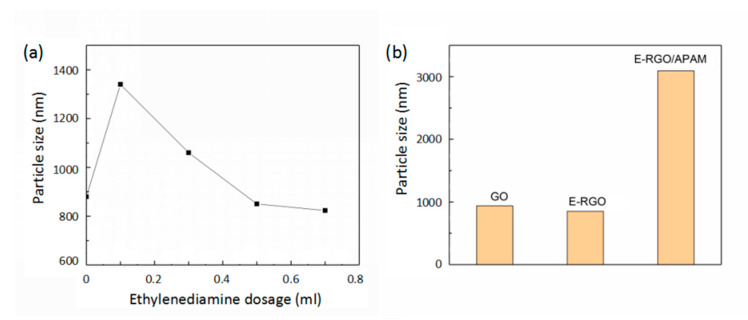
(**a**) ethylene diamine-reduced graphene oxide (E-RGO) particle size of different ethylenediamine dosage; (**b**) particle size of graphene oxide (GO), E-RGO and ethylene diamine-reduced graphene oxide/anionic polyacrylamide (E-RGO/APAM).

To reveal agglomeration capability of the reduced graphene oxide (RGO), the particle size of ethylenediamine-reduced graphene oxide (E-RGO) with different ratio of ethylenediamine (ED) was examined, as shown in Figure 1a. The particle size of graphene oxide (GO) was 880 nm; that of E-RGO increased as the amount of ED was 0.1 mL. With low doses of ED, the GO was mainly affected by the reduction, which led to a large decrease in the surface functional groups of the GO. Hence, the agglomeration capability was enhanced, and the particle size was increased. With the increase of the amount of ED, the particle size of E-RGO exhibited a reduced tendency, and lowered than the particle size of the GO when the amount of ED was above 0.5 mL, as expected [32]. It was indicated that the GO had a reduced agglomeration ability after reduction and favorable dispersion stability in water, which confirmed that the GO was modified by ED while being reduced, and that the grafting of the amino functional group hindered the agglomeration of the RGO. Hereinafter, the formulation of E-RGO/APAM was 0.5 mL for ED and 3:1 for GO:APAM unless otherwise specified.

Figure 1b shows the particle size of GO, E-RGO and E-RGO/APAM after ultrasonic dispersion in water. It was found that the particle size of E-RGO/APAM was the largest, which indicated that anionic polyacrylamide (APAM) can flocculate the dispersed graphene oxide in water to form a stable floc. The particle size of E-RGO/APAM was as large as three micrometers, so that E-RGO/APAM can be separated from water by conventional methods such as filtration and centrifugation. Therefore, secondary pollution caused by residual in water can be avoided, and it is suitable for use as an adsorbent material.

#### 3.1.2. Analysis of Infrared Spectrum and Raman Spectrum

**Figure 2:**
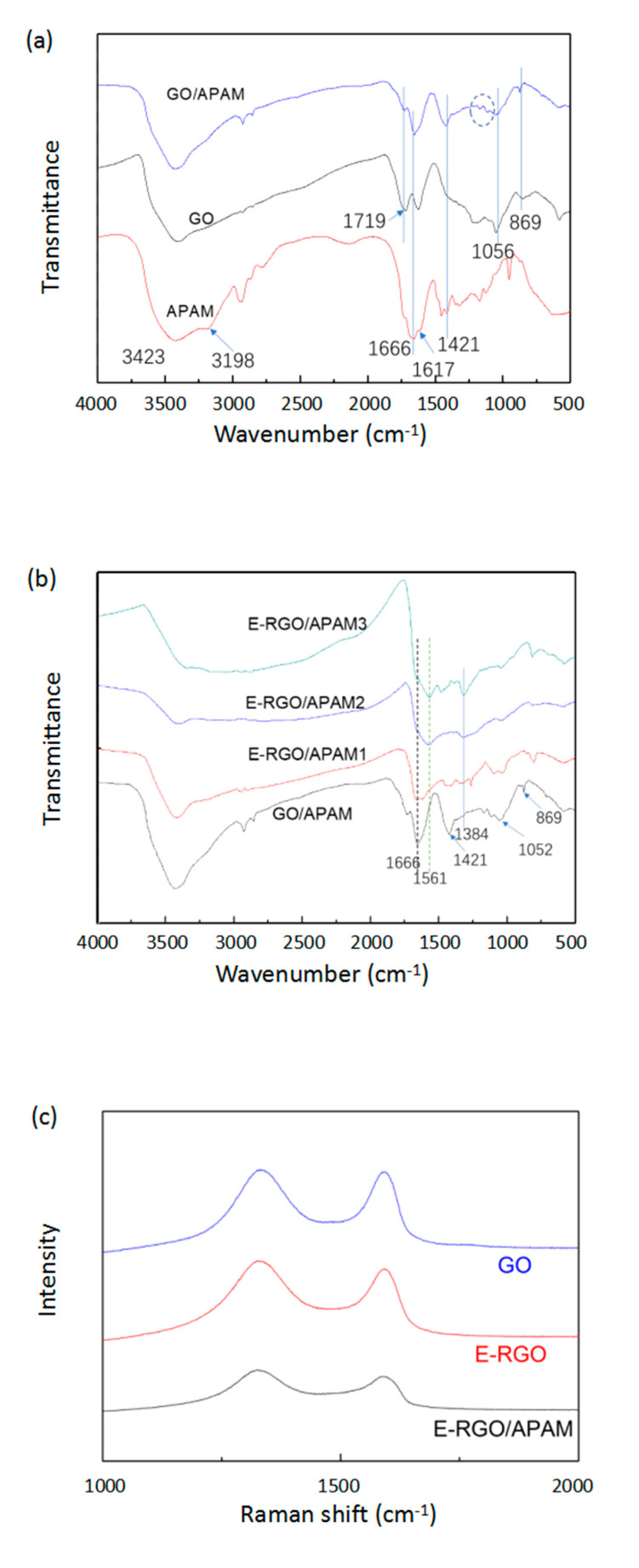
(**a**) FT-IR spectrum of APAM, GO, GO/APAM; (**b**) FT-IR spectrum of E-RGO/APAM with ethylenediamine dosage of (1–3) 0.1, 0.5, 1.0 mL, respectively; (**c**) Raman scattering spectrum of GO, E-RGO, E-RGO/APAM.

The successful incorporation of GO/APAM complex could be further confirmed by FT-IR spectrophotometer, and it was used to monitor the variation of chemical compositions as shown in Figure 2a. For the APAM, the peaks at 3423 cm^−1^ and 3198 cm^−1^ were due to–NH_2_ stretching vibrations, the peak at 1666 cm^−1^ was the characteristic absorption peak of the carbonyl group. The peak at 1617 cm^−1^ was due to the amide II (N–H) bending vibration [28,33]. What is more, the peaks at 1178 cm^−1^ and 1119 cm^−1^ were associated with C–N stretching vibration [33]. For the GO, the peaks at 1719 cm^−1^ and 1056 cm^−1^ were assigned to carbonyl and C–O–C characteristic peak [33]. Compared with the FT-IR spectrum of GO and APAM, the GO/APAM complex contains not only carbonyl (C=O) and C–O–C absorption peaks of GO, but also amide group and C–N absorption peaks of APAM [27]. For the FT-IR analysis, all results indicated that the interaction between GO and APAM occurred, successfully as proposed.

The FT-IR spectra of E-RGO/APAM with different ratio of ethylenediamine (ED) in the solution are shown in Figure 2b. With the increase of ED dosage, the carbonyl group exhibited a reduced tendency, the peak at 1720 cm^−1^ disappeared immediately and the peak at 1052 cm^−1^ weakened and eventually disappeared. The characteristic peak at 3198 cm^−1^ which were assigned to–NH_2_ of APAM disappeared and the intensity of the characteristic peak of the amide group was weakened (1668 cm^−1^ and 1617 cm^−1^), which may be due to the degradation of APAM in high temperature and alkaline conditions. Moreover, E-RGO/APAM exhibited the peak at 1561 cm^−1^, which was due to N–H stretching vibration and the peak at 1384 cm^−1^, which was due to C–N stretching vibration. The broad band between 1255 cm^−1^ and 1645 cm^−1^ was the joint effect of C–N stretching vibration and the out-of-plane N–H and–NH_2_ [16]. Above all, these results confirmed that ED was functionalized onto the GO successfully.

The Raman spectra of GO, E-RGO (ethylene diamine dosage is 0.5 mL) and E-RGO/APAM (ethylene diamine dosage is 0.5 mL) are shown in Figure 2c. It can be seen that E-RGO and E-RGO/APAM all have significant absorption peaks at 1332 cm^−1^ and 1590 cm^−1^, which were denoted as D peak and G peak, respectively. This indicated that APAM combines well with E-RGO. Generally, the intensity ratio of D peak to G peak (ID/IG) is used to analyze the order of crystal structure and grain size. The higher ID/IG value means that the structure of the graphene is more disordered and the greater degree of aggregation [34,35]. It can be calculated that the ID/IG value of GO was 0.95 and the ID/IG value of E-RGO and E-RGO/APAM were 1.11 and 1.13, respectively. It was revealed that the ID/IG value increased after compounding with the APAM, which was due to the APAM can be inserted into the RGO slices and the spacing between the slices increased.

#### 3.1.3. Morphology Analysis

**Figure 3:**
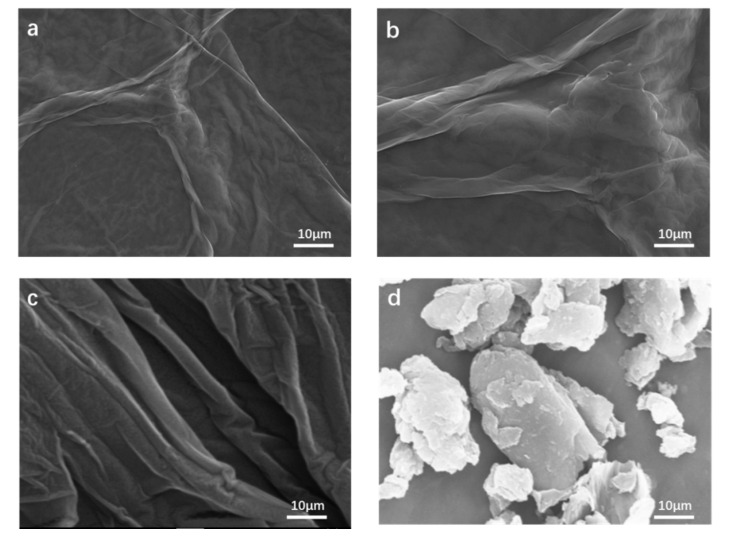
(**a**,**b**) SEM image of graphene oxide; (**c**) SEM image of E-RGO; (**d**) SEM image of E-RGO/APAM.

The surface morphologies of the various samples were observed by a scanning electron microscope (SEM) as shown in Figure 3. It can be seen that the surface of the GO substrate had wrinkles and exhibited a multilayered structure, but most of it was smooth and dense relatively, mainly due to the interaction of oxygen-containing functional groups. The surface morphology of E-RGO had more wrinkles and the surface became rough, which was because ED prevented the occurrence of overlapping agglomeration between sheets during the grafting of amino groups on GO. It can be seen that the surface of the composite E-RGO/APAM was uneven and had many protrusions. This surface topography was attributed to the high flocculation of APAM. It was revealed that APAM prevented agglomeration and effectively increases the composite, which was due to the APAM can be inserted into the RGO and leading to the formation of such a surface. Consequently, E-RGO and E-RGO/APAM had more adsorption sites for removing Pb(II) ion and were expected to have better adsorption effects.

### 3.2. Characterization of Pb(II) Adsorption Properties of E-RGO/APAM

**Figure 4:**
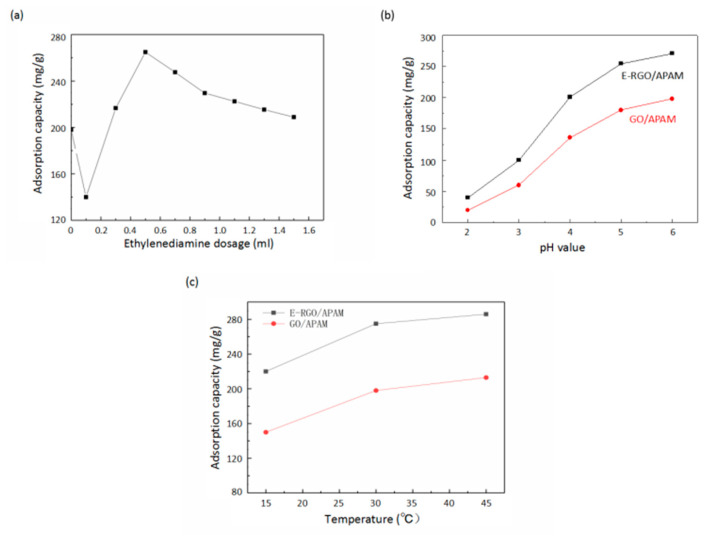
(**a**) Effect of ethylenediamine dosage on adsorption capacity of E-RGO/APAM; (**b**) effect of pH value on adsorption capacity of GO/APAM and E-RGO/APAM; (**c**) effect of temperature on adsorption capacity of GO/APAM and E-RGO/APAM.

The adsorption capacity of the adsorbent for Pb(II) prepared by different ratio of ethylenediamine (ED) is shown in Figure 4a. Without ED, the adsorption capacity of Pb(II) adsorbed by GO/APAM was 198.7 mg/g. With low dose of ED, the adsorption capacity of Pb(II) adsorbed by E-RGO/APAM was lower than that of GO/APAM, which was mainly due to the reduction of GO when the amount of ED was few. After GO was reduced, the surface oxygen-containing functional groups decreased, resulting in the decrease of the active sites and the weakening of adsorption capacity. It was indicated that with the increase of the amount of ED, the adsorption capacity of Pb(II) adsorbed by E-RGO/APAM was enhanced, which was mainly due to the dominant effect of ED on RGO modification and the introduction of reactive groups such as amino group. The chelating ability of the amino group to Pb(II) is stronger than that of the oxygen-containing functional groups such as carboxyl group, thus the adsorption ability was enhanced. It was revealed that with the further increase of ED, the amount of adsorbed Pb(II) gradually decreases. This was mainly due to the rapid degradation of APAM under alkaline condition, which reduced the adsorption capacity of APAM in the solution. On the other hand, ED regains its dominant position in the reducibility of GO, and the active groups massively disappear, which led to a decrease in adsorption capacity. It was suggested that the strongest adsorption capacity was appeared as the amount of ED was around 0.5 mL.

The adsorption capacity of the adsorbent for Pb(II) with different pH value is presented in Figure 4b. The pH value of the solution has an important influence on the adsorption performance, because GO, APAM and RGO contain functional groups that have electrostatic interaction or complexation with Pb(II), such as–COOH,–OH and–NH_2_. This fact was attributed to the H^+^ concentration in the solution increased while the pH value decreased, the groups such as–NH_2_ and–COOH were protonated and the electrostatic repulsion between the adsorbent and Pb(II) was increased, which led to the loss of capacity to adsorb Pb(II). It was indicated that the strongest adsorption capacity was appeared as the pH value was 6.0. As seen in Figure 4b, E-RGO/APAM had a stronger adsorption capacity than GO/APAM at the same pH value.

Figure 4c shows that the effect of different adsorption temperatures on the ability of E-RGO/APAM to adsorb Pb(II). It can be seen that with the increase of temperature, the adsorption capacity of E-RGO/APAM to Pb(II) increased, which was indicated that the adsorption process was an endothermic process and the increase of temperature was beneficial to overcome the energy barrier between Pb(II) and E-RGO/APAM. Overall, rose in temperature and elevated pH value within a certain range contributed to strengthen the adsorption capacity.

### 3.3. Adsorption Kinetics and Adsorption Isotherm

**Figure 5:**
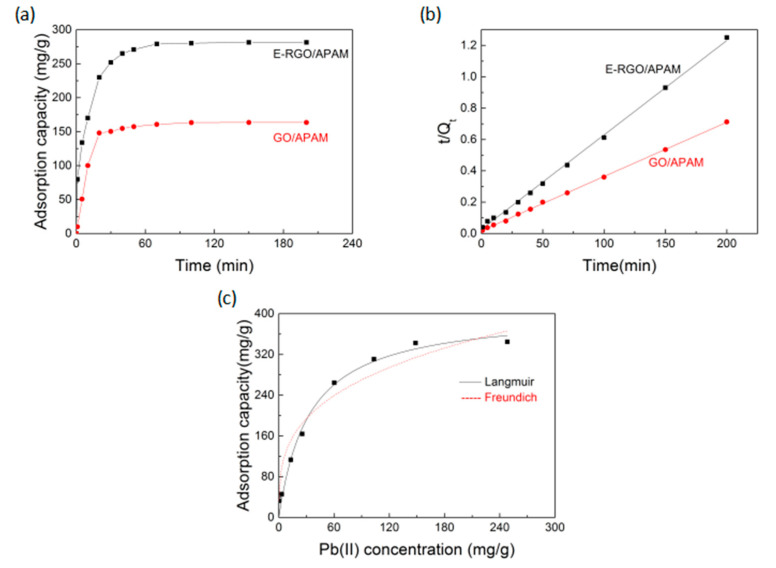
(**a**) Influence of adsorption time on the adsorption capacity of GO/APAM and E-RGO/APAM; (**b**) pseudo second order kinetic equation fitting of Pb(II) adsorption by GO/APAM and E-RGO/APAM; (**c**) Langmuir and Freundlich adsorption model of E-RGO/APAM (conditions: pH = 5, *T* = 30 °C).

Adsorption kinetics is an important indicator to study the adsorption capacity of adsorbent, which is used to describe the adsorption rate of adsorbent. As seen from the adsorption process in Figure 5a, the amount of Pb(II) adsorbed by GO/APAM increased rapidly in the first 20 min, then the growth slowed down and reached the adsorption equilibrium at about 50 min; the adsorption amount of Pb(II) at the equilibrium of adsorption was 160.05 mg/g. The amount of Pb(II) adsorbed by E-RGO/APAM exhibited a rapidly increased tendency in the first 40 min and after reaching the adsorption equilibrium in 70 min, the adsorption amount no longer further increased with time and the adsorption amount of Pb(II) at the equilibrium of adsorption was 281.07 mg/g. Compared with GO/APAM, the equilibrium adsorption capacity of E-RGO/APAM was obviously strengthened, which was mainly due to the fact that the utilizing of ED introduced reactive amino group and increased the surface area of the adsorbent.

In order to further study the adsorption process, the experiment was fitted using a pseudo second order kinetic equation and the equation [36] is expressed as:

(5) $$\frac{d_{Q_{t}}}{d_{t}}=K_{2}{\left(Q_{e}-Q_{t}\right)}^{2}$$

where *t* (min) is the adsorption time, *Q_e_* and *Q_t_* are the adsorption capacity (mg/g) at the time of adsorption equilibrium and at time *t*, *K_2_* (g/mg min) is the second order kinetic velocity constant. Integrate the expression to get the following equation:

(6) $$\frac{t}{Q_{t}}=\frac{1}{K_{2}Q_{e}^{2}}+\frac{t}{Q_{e}}$$

Use Equation (6) for *t/Q_t_~t* curve. This is presented in Figure 5b and the relevant parameters calculated are shown in Table 1. It can be found that the experimental data of the two adsorbents and the fitted straight line had a high degree of coincidence, which indicated that the adsorption process was mainly based on chemical adsorption and was controlled by the adsorption rather than diffusion. The adsorption kinetics *Q_e_* of the GO/APAM and E-RGO/APAM were 168.3 mg/g and 290.6 mg/g, respectively. The calculated *Q_e_* value was very close to the experimental data, according to pseudo-second-order kinetic mode. It was indicated that E-RGO/APAM had a higher adsorption capacity than GO/APAM and the utilizing of ED significantly strengthened the adsorption capacity.

The adsorption isotherm is used to describe the interaction between the adsorbate and the adsorbent and refers to the relationship between the adsorbate and the concentration in the adsorbent when the adsorption process reaches equilibrium. In most cases, the study is carried out under constant temperature and adsorption equilibrium conditions. The most commonly used adsorption models are the Langmuir and Freundlich equation [2,37]. The Langmuir adsorption model is used to simulate the adsorption process as a single layer of uniform adsorption, assuming no interaction between the molecules. The most basic feature is that the adsorption amount in the initial stage increases with the increase of the equilibrium concentration, and then the growth slows down and the final adsorption amount tends to a stable value. The Langmuir equation can be described by the following equation:

(7) $$\frac{1}{Q_{e}}=\frac{1}{K_{L}Q_{m}C_{e}}+\frac{1}{Q_{m}}$$

where *Q_e_* (mg/g) is the adsorption amount of adsorption equilibrium, *Q_m_* (mg/g) is the maximum adsorption amount, *C_e_* (mg/L) is the initial equilibrium concentration of the adsorbate and *K_L_* (L/mg) is the adsorption constant.

The Freundlich adsorption model is a semi-empirical model and is an adsorption model in the case where the surface of the adsorbent is not uniform. The model is used to describe the nonuniform adsorption process, which does not form a straight line at low concentration and does not tend to a stable value at high concentration. The Freundlich equation can be described by the following equation:

(8) $$\ln Q_{e}=\ln K_{F}+\frac{1}{n}\ln C_{e}$$

where *K_F_* is the Freundlich constant, which represents the amount of adsorption, and *n* indicates the adsorption strength.

It can be seen from Figure 5c that the adsorption amount of E-RGO/APAM increased as the concentration of Pb(II) increased. The analysis results of the Langmuir and Freundlich adsorption models were shown in Table 2. Compared with the Freundlich adsorption model, the Langmuir adsorption model had a better correlation, *R^2^* = 0.984, which indicated that E-RGO/APAM adsorbed Pb(II) mainly by chemisorption and was dominated by monolayer adsorption. It was attributed to the active sites are distributed evenly on the surface of E-RGO/APAM. The maximum adsorption capacity was calculated to be 400.8 mg/g by using the Langmuir adsorption model and the Freundlich adsorption model showed that the value of 1/n was 0.3187, which was less than 0.5, indicated that the adsorption process easily occurred. The results showed that E-RGO/APAM is a very potential adsorbent for Pb(II) ions.

## 4. Conclusions

A facile and versatile approach for preparation of adsorbent is described based on the strong interactions between APAM and RGO for enhancing adsorption performance. In order to adsorb Pb(II) in aqueous solution without secondary pollution, E-RGO/APAM, with a large quantity of adsorption sites and strong flocculation was prepared. The active adsorption sites and adsorption capacity of the absorbent were significantly improved was confirmed by laser Raman scattering spectroscopy and methylene blue test adsorption method. Static adsorption experiment showed that the dosage of ethylenediamine, the pH value and the temperature of the solution have an important influence on the adsorption performance. Moreover, the process of adsorption of Pb(II) by E-RGO/APAM can be fitted by pseudo second order kinetic equation, indicating that the adsorption rate of the adsorbent depends on the chemisorption process, and the theoretical maximum adsorption amount of E-RGO/APAM is 400.8 mg/g. Therefore, this research demonstrates a deeper understanding of the interactions between E-RGO/APAM and Pb(II), which can provide great inspiration for the design and development of adsorbent.

## Figures and Tables

**Table 1:** Adsorption capacity (Q_e_), second order kinetic velocity constant (k_2_) and linear relationship (R^2^).

	Pseudo Second Order Kinetic Equation		
	$\frac{t}{Q_{t}}=\frac{1}{K_{2}Q_{e}^{2}}+\frac{t}{Q_{e}}$		
	*Q*_e_	*k*_2_	*R* ^2^
E-RGO/APAM	290.6 mg/g	0.0017	0.9996
GO/APAM	168.3 mg/g	0.0024	0.9982

**Table 2:** Maximum adsorption capacity (*Q_m_*), Langmuir adsorption constant (*K_L_*), adsorption strength (*n*) and Freundlich adsorption constant (*K_F_*), linear relationship (*R^2^*).

Langmuir Adsorption Model			Freundlich Adsorption Model		
$Q_{m}$	$K_{L}$	$R^{2}$	1/n	$K_{F}$	$R^{2}$
400.8 mg/g	0.030	0.984	0.3187	69.55	0.948
